# Role of Circular RNA in Kidney-Related Diseases

**DOI:** 10.3389/fphar.2021.615882

**Published:** 2021-03-11

**Authors:** Xin-Tian Chen, Zhong-Wei Li, Xue Zhao, Min-Le Li, Ping-Fu Hou, Su-Fang Chu, Jun-Nian Zheng, Jin Bai

**Affiliations:** ^1^ Cancer Institute, Xuzhou Medical University, Xuzhou, China; ^2^ Center of Clinical Oncology, Affiliated Hospital of Xuzhou Medical University, Xuzhou, China

**Keywords:** circRNAs, renal cell carcinoma, acute kidney injury, chronic kidney diseases, EMT

## Abstract

The kidney is vital in maintaining fluid, electrolyte, and acid–base balance. Kidney-related diseases, which are an increasing public health issue, can happen to people of any age and at any time. Circular RNAs (circRNAs) are endogenous RNA that are produced by selective RNA splicing and are involved in progression of various diseases. Studies have shown that various kidney diseases, including renal cell carcinoma, acute kidney injury, and chronic kidney disease, are linked to circRNAs. This review outlines the characteristics and biological functions of circRNAs and discusses specific studies that provide insights into the function and potential of circRNAs for application in the diagnosis and treatment of kidney-related diseases.

## Background

Circular RNAs (circRNAs) were first discovered in RNA viruses in the 1970s. They were used to be considered byproducts of mis-splicing and much rare (Sanger et al., 1976). With the development of next-generation sequencing and bioinformatic analysis, circRNAs have been recognized to be widely found, and have their own biological functions in the pathogenesis of various diseases (Hansen et al., 2013; Zhang et al., 2020).

The kidney plays an important role in maintaining water and electrolyte balance, and regulating homeostasis. Renal diseases, including renal cell carcinoma (RCC), acute kidney injury (AKI), chronic kidney disease (CKD), are major causes of kidney failure, which leads to a poor quality of life of patients and poses great burden and loss to the society (Lowenstein and Grantham, 2017).

circRNAs are dynamically expressed and spatiotemporally regulated in kidney-related diseases, this review summarizes the formation and characteristics of circRNAs and discusses how they are involved in the progression of these disorders to propose circRNAs as optional strategies for regulating disease progression and improving therapeutic outcomes.

## Formation and Characteristics of Circular RNAs

RNAs can be classified into protein-coding or nonprotein-coding molecules according to their size, location and function. Only about 2% of RNAs are protein coding, and most RNAs belong to noncoding RNAs (ncRNAs), which are a kind of RNA with large amount and diverse functions. These RNA molecules can be sorted in terms of their sizes, and 200 nucleotides can separate small ncRNAs from long ncRNAs (lncRNAs). MicroRNAs (miRNAs), which have 20–22 nucleotides and can downregulate the expression of target protein-coding genes, are among the most well-researched small ncRNAs. lncRNAs are generally divided into linear lncRNAs (acquiesced as lncRNAs) and circular RNAs (circRNAs) (Brandenburger et al., 2018).

**FIGURE 1:**
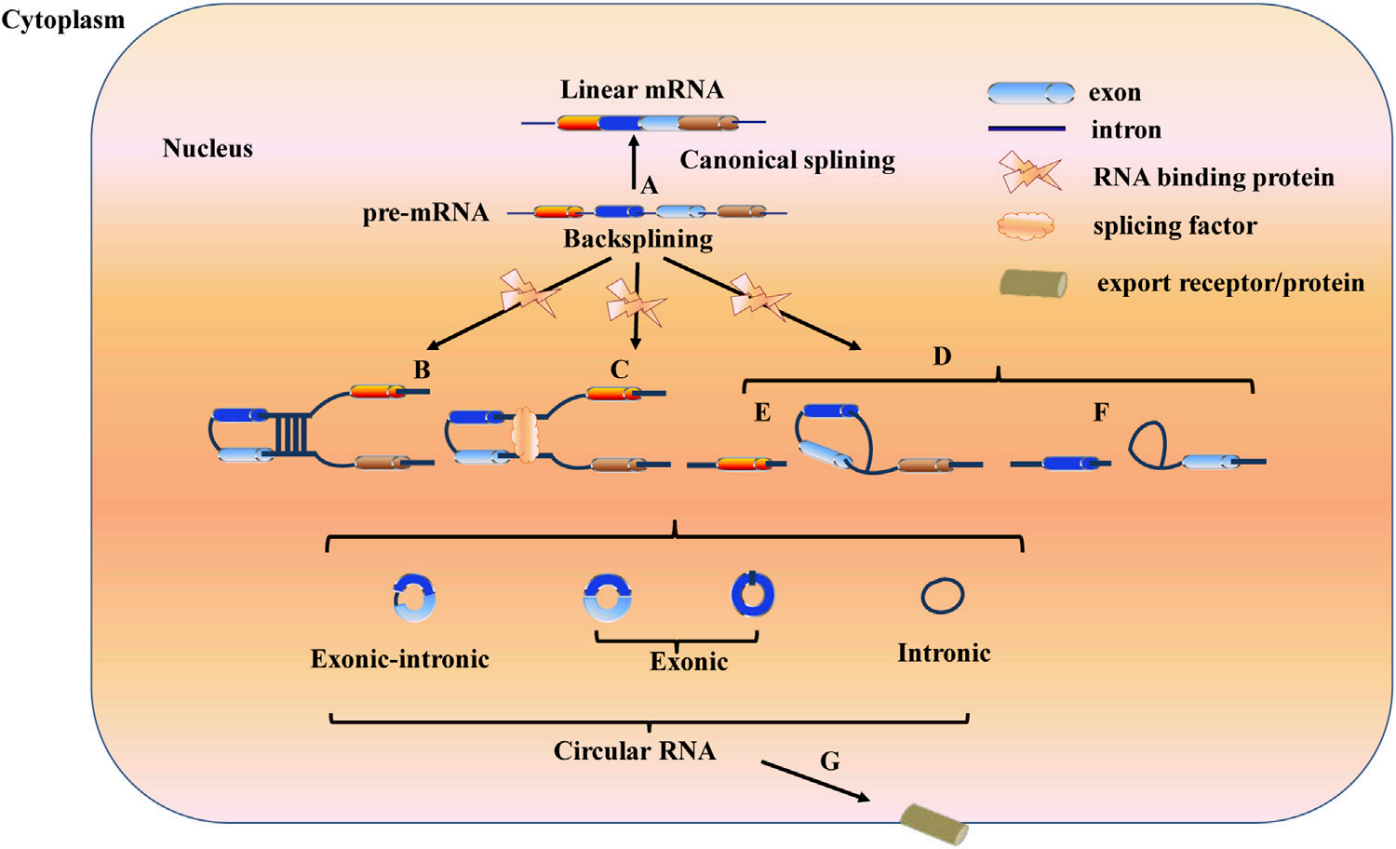
Three mechanisms of circRNAs biogenesis Canonical splicing results in the formation of mature mRNA after the removal of introns **(A)**; While back-splicing contributes to the formation of circRNAs. Intron pairing-driven circularization, RNA-binding protein (RBP)-driven circularization and lariat-driven circularization are three mechanisms to generate circRNAs. In intron pairing-driven circularization the back-splicing event is guided by base pairing of complementary sequences located in the introns flanking the back-spliced exons **(B)**. In RNA-binding protein (RBP)-driven circularization, RBPs play a dominant role, they recognize and binding to specific motifs located in the introns flanking the circularized exons **(C)**. In lariat-driven circularization, including intronic lariats and exon-containing lariats circularization, circRNAs are formed during linear splicing **(D)**. Intronic lariats circularization: circRNAs are formed when an intron is removed during precursor mRNA (pre-mRNA) splicing **(F)**. Exon-containing lariats: circRNAs are generated during exon-skipping **(E)**. Those circRNAs then are exported to cytoplasm through export receptor or export protein including Hel25E in *drosophila* and UAP56/URH49 in human **(G)**.

Most precursor messenger RNAs (pre-mRNAs) are spliced into linear RNA molecules through canonical splicing. However, pre-mRNAs can also be spliced into circRNAs through back splicing. A circRNA is a closed loop structure formed through the covalent bonding of 5′-cap structures and 3′-poly A tails. According to different splicing sources, circRNAs can be divided into exonic circRNAs (ecircRNAs), circular intron circRNAs (ciRNAs), and exon- and intron-derived or retained intron circRNAs (EIciRNA) (Han et al., 2017). Three circularization mechanisms, namely, intron pairing-driven, RNA-binding protein-driven, and lariat-driven mechanisms, are known to achieve the formation of circRNAs (Aufiero et al., 2019) (Figure 1).

circRNAs have the following highlighted characteristics. 1) They are widespread and stable. Many circRNAs are highly conserved in different species, including humans, mice, *Drosophila*, and yeasts (Cocquerelle et al., 1993; Jeck et al., 2013; Wang et al., 2014). The expression patterns of circRNAs vary in different tissues and conditions. They are enriched in the brain and kidneys, and are abnormally expressed when diseases occur. They can be detected in the blood, urine, saliva, and also in exosomes (Salzman et al., 2012; Li et al., 2015). circRNAs are resistant to RNase, benefiting from their covalently closed loop structures. This special structure gives circRNAs higher expression and longer average half-life than that of their linear isomers (Yan et al., 2019). This feature lays the foundation of circRNAs as biomarkers and regulators. 2) circRNAs have specific location and expression. ecircRNAs, which account for the majority of circRNAs, are mostly located in the cytoplasm. ecircRNAs can act as miRNA sponges, which prevent miRNAs from forming a complementary pair with target mRNA 3ʹ-UTRs; as a result, the expression of target mRNAs increases. ecircRNAs also can stabilize or activate the functions of miRNAs, decreasing the expression of mRNAs, which are called miRNA reservoirs (Hansen et al., 2013; Memczak et al., 2013; Qu et al., 2015; Zheng et al., 2017; Wang et al., 2018). Conversely, ciRNAs and EIciRNAs are mainly located in the nucleus and may be involved in the regulation of gene expression at transcriptional or post-transcriptional levels (Jeck et al., 2013; Zhang et al., 2013; Han et al., 2017).

There are some computational tools and databases are designed to identify and analyze circRNAs (Tables 1, 2). These predictive tools can be utilized to identify different kinds of circRNAs based on different identification strategies. Appropriate tools should be selected on the basis of the purposes of a particular research, and multiple predictive tools are recommended to reduce the likelihood of losing target circRNAs (Aufiero et al., 2019; Shang et al., 2019).

**TABLE 1:** Analysis tools of circRNAs.

Name (Years)	Description of the tools	Website address	References
*CIRI-AS (2015)*	Detecting circRNAs from transcriptome data	https://sourceforge.net/projects/ciri/	(Gao et al., 2015)
*Acfs (2015)*	Discovering circRNAs from RNA-Seq data	https://github.com/arthuryxt/acfs	(You et al., 2015)
*PTESFinder (2016)*	A computational method to identify post-transcriptional exon shuffling (PTES) events	http://ibi.zju.edu.cn/bioinplant/tools/manual.htm	(Izuogu et al., 2016)
*UROBORUS (2016)*	An efficient tool that can detect circRNAs with low expression levels in total RNA-seq without RNase R treatment	http://uroborus.openbioinformatics.org/en/latest/	(Song et al., 2016)
*Sailfish-cir (2017)*	Quantification the expression of circRNAs from high-throughput RNA-seq data	https://github.com/zerodel/Sailfish-cir	(Finucane et al., 1999)
*CircPro (2017)*	An integrated tool for the identification of circRNAs with protein-coding potential	http://bis.zju.edu.cn/CircPro	(Meng et al., 2017)
*CircMarker (2018)*	A fast and accurate algorithm for circular RNA detection based on k-mer analysis	https://github.com/lxwgcool/CircMarker	(Li et al., 2018b)

**TABLE 2:** Online circRNA databases.

Name (Years)	Description of the database	Website address	References
*Circ2Traits (2013)*	A comprehensive knowledgebase of potential association of circular RNAs with diseases in human	http://gyanxet-beta.com/circdb/	(Hancock, 2014)
*circBase (2014)*	Merged and unified datasets of circRNAs. It provides scripts to identify known and novel circRNAs in sequencing data	http://www.circbase.org/	(Glažar et al., 2014)
*Circbank (2014)*	A comprehensive database of human circRNA.	http://www.circbank.cn/	(Glažar et al., 2014)
*Starbase v2.0 (2014)*	Database of circRNA-miRNA interactions	http://starbase.sysu.edu.cn/index.php	(Li et al., 2014)
*Circnet (2015)*	A database that provides tissue-specific circRNA expression profiles and circRNA-miRNA-gene regulatory networks	http://circnet.mbc.nctu.edu.tw/	(Liu et al., 2016)
*Circinteractom (2015)*	Mapping RNA-binding proteins and miRNA-binding sites on human circRNAs	https://circinteractome.irp.nia.nih.gov/	(Dudekula et al., 2016)
*Deepbase v2.0 (2016)*	Identification, expression, evolution and function of small RNAs, LncRNAs and circular RNAs from deep-sequencing data	http://deepbase.sysu.edu.cn/	(Zheng et al., 2016)
*CircRNADb (2016)*	Including the detailed information of the circRNA, and provides the function of data search, browse, download, submit and feedback to study particular circular RNA.	http://reprod.njmu.edu.cn/circrnadb	(Chen et al., 2016)
*TSCD (2017)*	Human and mouse tissue-specific (TS) circRNAs	http://gb.whu.edu.cn/TSCD/	(Xia et al., 2017)
*CSCD (2018)*	A cancer-specific circRNA database	http://gb.whu.edu.cn/CSCD/	(Xia et al., 2018)
*Circpedia v2 (2018)*	An updated database for comprehensive circRNA annotation from over 180 RNA-seq datasets across six different species	http://www.picb.ac.cn/rnomics/circpedia/	(Zhang et al., 2016)
*CircRNADisease (2018)*	Each entry in the circRNADisease includes detailed information on a circRNA-disease association	http://cgga.org.cn:9091/circRNADisease/	(Zhao et al., 2018)
*ExoRBase (2018)*	A database of circRNA, lncRNA and mRNA in human blood exosomes	http://www.exoRBase.org	(Li et al., 2018a)

## Circular RNAs in Kidney Development

The role of circRNAs in the development of the brain and neurodegenerative disorders have already been expounded (Westholm et al., 2014; Huang et al., 2018; Mehta et al., 2020), while theirs role in kidney development and diseases still need to be clarified. In 1996, the first kidney-related circRNA known as cytochrome P450 2C24 gene was found in rat kidney; it is a transcript containing exons 2 and 4 spliced at the correct sites, but the donor site of exon 4 is directly joined to the acceptor site of exon 2 (exon scrambling) (Zaphiropoulos, 1996). After 2 years, a circular formin mRNA transcript was found in the brain and kidney of mice; the blunting of circRNA formation leads to renal aplasia in gene-targeted mutant mice, suggesting that circRNAs may also play a critical role in the development of the kidney of mice (Chao et al., 1998). RNA sequencing analysis in humans has verified that the expression of 1,664 circRNAs in fetal kidney samples is higher than that in the corresponding adult kidney tissues. Further analysis has shown that about 474 circRNAs are more enriched in the kidney than in other organs, indicating the potentially critical roles of circRNAs in the development of the kidney of humans (Xu et al., 2017). Existing studies only focus on the changes of circRNAs expressions, further studies should pay attention to the specific mechanisms of how circRNAs work during kidney development.

## Circular RNAs in Renal Cell Carcinoma

RCC is one of the most common malignant cancers in the world, although the 5-years survival rates have shown some considerable improvements, the overall prognosis is still far from satisfactory (Siegel et al., 2017). The possible mechanism on how RCC deteriorates and some specific biomarkers should be defined to optimize treatment strategies.

**FIGURE 2:**
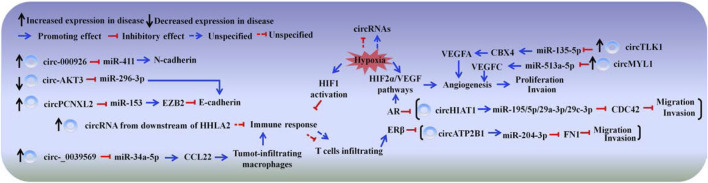
CircRNAs participate in renal cell carcinoma. The schematic diagram depicts some of circRNAs in renal cell carcinoma progression.

The motility of tumor cells is often enhanced, with remodeling of tumor microenvironments when tumor develops. Dysregulated circRNAs are involved in RCC progression by altering tumor cell dynamics, participating in the remodeling of tumor microenvironments including matrix remodeling, hypoxia and immunesuppression and so on ((Figure 2; Table 3).

**TABLE 3:** The potential mechanisms and target genes of circRNAs in kidney diseases.

Kidney-related diseases	circRNAs	Target miRNA	miRNA targeted genes	Function
Acute kidney injury	has_circ_001334 (Kölling et al., 2019)	Undefined	Undefined	Biomarker in urine
	ciRs-126 (Kölling et al., 2018)	Undefined	Undefined	Biomarker in blood
	circ_0004153 (Cheng et al., 2019)	rnomiR-144-3p	Gpnmb Naglu	NF- κB activation Inflammation
	circ_0114427 (Cao et al., 2020)	miR-494	AFT3	NF- κB activation
	circ-AKT3 (Xu et al., 2020)	miR-144-5p	Wnt/β-catenin pathway	Wnt/β-catenin activation
	circ-YAP1 (Huang et al., 2020)	miR-21-5p	PI3K/AKT/mTOR pathway	PI3K/AKT/mTOR activation
	circ-VMA21 (Shi et al., 2020)	miR-9-3p	SMG1	Oxidative stress
Chronic kidney disease	circHLA-C (Luan et al., 2018)	miR-150	Undefined	Fibrosis-associated genes express
	circRNA_15698 (Hu et al., 2019)	miR-185	TGF-β1	Extracellular matrix-related protein synthesis
	circ_WBSCR17 (Li et al., 2020a)	miR-185-5p	SOX6	ECM accumulation
	circRNA_002453 (Ouyang et al., 2018)	Undefined	Undefined	Biomarker in blood
	circ_DLGAP4 (Bai et al., 2020)	miR-145	ERBB3/NF-κB/MMP-2 pathway	Inflammation
	circLRP6 (Chen et al., 2019)	miR-205	HMGB 1/TLR4/NF-κB pathway	Inflammation
	circRNA_010383 (Peng et al., 2020)	miR-135a	TRPC1	ECM accumulation
	circ-AKT3 (Tang et al., 2020)	miR-296-3p	E-cadherin	ECM accumulation
	circNr1h4 (Lu et al., 2020)	miR-155-5p	Far1	Reactive oxygen
	cANRIL (Deng et al., 2019)	miR-9	NF- κB and JNK/p38 pathways	Inflammation
Renal cell carcinom	has_circ_001895(Chen et al., 2020)	miR-296-5p	SOX12	Promoting proliferation, migration, invasion, inhibiting apotosis
	circEGLN3 (Lin and Cai, 2020)	miR-1299	IRF7	Promoting proliferation, migration, invasion, inhibiting apotosis
	circNRIP1 (Dong et al., 2020)	miR-505	AMPK and PI3KL/AKT/mTOR pathways	Promoting proliferation, migration, invasion, inhibiting apotosis
	circNUP98 (Yu et al., 2020)	miR-567	PRDX3	Promoting proliferation, migration, invasion, inhibiting apotosis
	has_circ_0072309 (Chen et al., 2019)	miR-100	PI3KL/AKT/mTOR	Inhibiting proliferation, migration, invasion, promoting apotosis
	circC3P1 (Chen et al., 2020)	miR-21/PTEN	PI3K/AKT and NF- κB	Inhibiting proliferation, migration, invasion, promoting apotosis
	circFNDC3B (Chen et al., 2020)	miR-99a	JAK1/STAT3 and MEK/ERK pathways	Promoting proliferation, migration
	circ-ZNF652 (Zhang and Guo, 2020)	miR-205	JAK1/STAT3 and MEK/ERK pathways	Promoting proliferation, migration
	circ-ZNF609(Xiong et al., 2019)	miR-138-5p	FOXP4	Promoting proliferation, invasion
	circDHX33 (Wang et al., 2020)	miR-498-3p	MERK	Promoting proliferation, invasion
	circHIPK3(Li et al., 2020)	miR-508-3p	CXCL13	Promoting proliferation, migration, invasion
	circUBAP2 (Sun et al., 2020)	miR-148a-3p	FOXK2	Promoting proliferation, migration, invasion
	circ_001842 (Zeng et al., 2020)	miR-502-5p	SLC39A14	Promoting proliferation, migration, invasion
	cRAPGEF5 (Chen et al., 2020)	miR-27a-3p	TXNIP	Inhibiting proliferation, migration
	has_circ_0001451 (Wang et al., 2018)	Undefined	Undefined	Inhibiting proliferation, promoting apotosis

### Tumorigenesis-Related Circular RNAs

circRNAs are widely involved in many biological processes including proliferation, invasion and apoptosis of RCC cells.

circRNAs can act as an miRNA sponge to accelerate RCC progression. hsa_circ_0002286/has-mir-222-5p/TRIM2 axis had been identified to play a critical role in the progression of RCC via database analysis (Wei et al., 2020). Hsa_circ_001895 is upregulated in RCC specimens; knocked down hsa_circ_001895 can inhibit RCC progression and promote apoptosis by reducing the adsorption of miR-296-5p and decreasing the expression of sex-determining region Y (SRY)-box 12 (SOX12) (Chen et al., 2020). SOX12 participates in cell differentiation during embryonic development, and its high expression predicts poor prognosis (Gu et al., 2018). Similarly, the high expression of interferon regulatory factor 7(IRF7) is related to a poor survival rate. circEGLN3 promotes the proliferation and aggressiveness of RCC via miR-1299-mediated IRF7 activation (Lan et al., 2019; Lin and Cai, 2020) The up-regulated circDHX33 can promote the proliferation and invasion of RCC, by sponging with miR-489-3p and increasing mitogen-activated protein kinase (MEK) expression (Wang et al., 2020). Likewise, forkhead-box P4 (FOXP4) highly contributes to cancer cell growth and invasion (Yang et al., 2015), circ-ZNF609 remarkably increases in RCC tissues and consequently promotes the expression of FOXP4 by sponging with miR-138-5p (Xiong et al., 2019). The high expressed circ_001842 was found to elevate SLC39A14 expression by binding to miR-502-5p, thereby promoting invasion, metastasis and inflammation of RCC (Zeng et al., 2020). Forkhead box K2 (FOXK2) belongs to the forkhead box transcription factor family and plays essential roles in cellular proliferation and survival (van der Heide et al., 2015). The decreased circUBAP2 sponges with miR-148a-3p to increasing FOXK2 expression to promote RCC progression (Sun et al., 2020). Additionally, methyltransferase-like 14 (METTL14) is positively correlated with the tumor suppressor gene PTEN. Bioinformatic analysis has demonstrated that circRNAs may act as an miRNA sponge that decreases the mRNA expression of METTL14. The mRNA of METTL14 likely regulates the mRNA expression of PTEN by changing its m6A RNA modification level; leading to RCC progresses (Wang et al., 2019).

circRNAs are involved in some signaling pathways to regulate RCC progression. circRNA nuclear receptor-interacting protein 1 (circNRIP1) is overexpressed in RCC tissues, and si-circNRIP1 in ACHN and CAKI-1 cells deactivates AMPK and PI3K/AKT/mTOR pathways, which are mediated by miR-505 (Dong et al., 2020). circFNDC3B is highly expressed in RCC tissues and attends to the modulation of RCC growth and metastasis by activating the pathways of JAK1/STAT3 and MEK/ERK(Chen et al., 2020). The increased circ-ZNF652 has the similar effect by sponging with miR-205 (Zhang and Guo, 2020). It is also found that circNUP98 is under the regulation of STAT3, and it functions as a sponge of miR-567 and promotes the expression of PRDX3 (an antioxidant protein, belongs to the peroxiredoxins), leading to RCC progression (Cox et al., 2009; Yu et al., 2020).

circRNAs also act as a suppressor gene. cRAPGEF5 is downregulated and can inhibit the progression of RCC by acting as a sponge of miR-27a-3p to upregulate TXNIP(Chen et al., 2020). The TXNIP gene serves as a suppressor during oxidative stress-induced renal carcinogenesis (Jiao et al., 2019). Likewise, hsa_circ_0001451 is downregulated in RCC, and its inhibition leads to OSRC-2 and 786-O cell proliferation and decreases apoptosis (Wang et al., 2018). Similarly, hsa-circ-0072309 is poorly expressed, and plays antitumor roles by blocking the PI3K/AKT/mTOR cascades in CAKI-1 and ACHN cell by targeting miR-100 (Chen et al., 2019). Another circRNA called circC3P1 is downregulated, and its overexpression in ACHN restrains NF-κB pathways (Zhong et al., 2018; Chen et al., 2020).

### EMT-Related Circular RNAs

The distinguishing feature of EMT is the functional loss of cell adherens junctions (Nieto et al., 2016; Xue et al., 2019). Adherens junctions are mainly composed of a transmembrane calcium-dependent glycoprotein named E-cadherin (also known as CDH1), which is considered a tumor suppressor maintaining integrity at local and tissue levels (Lecuit and Yap, 2015). Abnormal E-cadherin expression is often observed in RCC samples, and the loss of E-cadherin is considered as early carcinogenic event (Evans et al., 2007). circPCNXL2 is increased in RCC tissues, it sponges with miR-153 to up-regulate the expression of Zinc finger E-box-binding homeobox 2 (ZEB2), which can inhibit the expression of E-cadherin (Nam et al., 2012; Zhou et al., 2018). While, circ-AKT3 is stably downregulated in RCC tissues and negatively related to metastasis. circAKT3 functions as an miR-296-3p sponge to increase E-cadherin expression (Xue et al., 2019).

In addition to E-cadherin, N-cadherin (also known as cadherin 2, CDH2), which is a mesenchymal marker, is differently expressed in RCC and EMT progression (Behnes et al., 2012; Alimperti and Andreadis, 2015). circ_000926 is highly expressed and may function as an miR-411 sponge to upregulate CDH2 expression, thereby facilitating EMT progression and leading to poor prognosis (Zhang et al., 2019).

EMT is a complicated process that involves multiple transcription factors, and several important signaling pathways (Shang et al., 2019). Changes in the expression of CDH1 and CDH2 elicit a domino effect, and other factors are needed in EMT progression. Therefore, further studies should focus on whether other circRNAs are involved and determine how they cooperate with EMT transcription factors or pathways to accelerate or impede EMT in renal cancer.

### Hormone Receptor-Related Circular RNA

RCC is 1.7 times more common in men than in women (Lee et al., 2012). Gender difference in the development and prognosis of renal cancer has been considered in numerous investigations (Aron et al., 2008; Marchioni et al., 2017; Lughezzani et al., 2019).

Androgen receptor (AR) is a transcriptional regulator involved in many cellular functions in men and women (Heemers and Tindall, 2007). The expression of AR can be detected in 14.8–42% of RCCs, which have short lifetimes (Langner et al., 2004; Noh et al., 2013). CDC42, as a member of the Rho family, is overexpressed in a number of human cancers and can be activated in response to extracellular matrix; it functions as a molecular switch for cell migration and invasion (Stengel and Zheng, 2011; Ni et al., 2013). Wang et al. (2017) found that the 3ʹ-UTR of miR-195-5p/29a-3p/29c-3p targets CDC42 to suppress its protein expression. circHIAT1, which can be suppressed by AR, increases miR-195-5p/29a-3p/29c-3p stability by acting as an miRNA reservoir to partly reverse AR-enhanced RCC migration and invasion.

Besides AR, estrogen receptor beta (ERβ) is involved in RCC progression. Yu et al. (2013) reported that ERβ expression is much higher in RCC cell lines than in breast cancer cell lines, and estrogen-activated ERβ acts as a tumor suppressor in RCC. However, clinical data from the TCGA database confirmed that higher ERβ expression was related to poorer prognosis in patients with RCC; these data serve as powerful evidence to prove that ERβ can be an oncogene in RCC progression (Song et al., 2015; Yeh et al., 2015). Fibronectin 1 (FN1) is highly expressed in vascular endothelial cells and vascular smooth muscles and promotes angiogenesis and endothelial cell migration, thereby aggravating RCC (Waalkes et al., 2010; Steffens et al., 2012). It is reported that ERβ-suppressed circATP2B1 functions as an miR-204-3p reservoir, it leads to miR-204-3p reduction, which increases FN1 expression and enhances RCC cell invasion (Han et al., 2018).

### Hypoxia- and Immune-Related Circular RNAs

Mutations in the VHL gene and abnormal angiogenesis in RCC can lead to an increased activity of hypoxia-induced factors (HIFs), which can activate the transcription of downstream oncogenes that contain anoxic response elements (HREs) and signal pathways to affect the proliferation and metastasis of cancer cells (Min et al., 2002; Hsieh et al., 2017; Shan et al., 2018).

Vascular endothelial growth factor (VEGF) is needed for angiogenesis (Carmeliet and Jain, 2000). circTLK1 is found over-expressed in RCC and correlated with poor prognosis. circTLK1 sponges with miR-136-5p to increase CBX4 (a small ubiquitin-related modifier E3 ligase) expression, which promoting the expression of VEGFA (Ismail et al., 2012; Li et al., 2020). The increased circMYLK also can capture miR-513a-5p to facilitate VEGFC expression (Li et al., 2020). The uncontrolled expression of VEGF fails to abnormal vascular structure, resulting in hypoxia and may participate in RCC metastasis progression further. For example, the loss of E-cadherin in the EMT of RCC is mainly due to HIF-1 activation, and AR promotes RCC progression mainly through the HIF2a/VEGF pathway (He et al., 2014).

RCC has been well recognized as a disease that can evade the immune system and strongly respond to immunotherapy (Perez-Ruiz et al., 2019). T cells and natural killer cells are the most common types of immune cells in RCC tumors; infiltrating T cells can promote RCC cell invasion by increasing ERβ expression (Van den Hove et al., 1997; Yeh et al., 2015). Likewise, tumor cells and tumor-infiltrating macrophages (TAMs) produce the chemokine CCL22, which attracts regulatory T cells (Tregs) to create an immune-suppressive microenvironment, thereby impairing anticancer immunity (Martinenaite et al., 2016). circ_0039569 is upregulated, it can promote RCC progression by upregulating CCL22 expression though sponging with miR-34a-5p (Jin et al., 2019). Human endogenous retrovirus-H long terminal repeat-associating protein 2 (HHLA2) can interact with PD-1 and CTLA-4, resulting in T cell suppression. A high HHLA2 expression in RCC tissues is associated with poor prognosis. Comprehensive microarray analysis identify that thousands of circRNAs, which are considered downstream of HHLA2 may function in immune response to participate in RCC progress (Minárik et al., 2013; Chen et al., 2019).

These results suggest that different circRNAs play multifaceted roles in RCC. Whether other hypoxic- and immune-related circRNAs are formed in RCC is still unknown, future studies may focus on finding new related circRNAs and functionally investigate how circRNAs work in a systematic network of deterioration in RCC.

## Circular RNAs in Acute Kidney Injury

AKI is characteristic as abrupt or rapid decline in the glomerular filtration rate, and mainly caused by ischemia/reperfusion (I/R), nephrotoxicity, and sepsis (Mehta et al., 2007; Barrantes et al., 2008). AKI is often diagnosed on the basis of creatinine levels, but creatinine assessments sometimes cannot accurately identify kidney function and it is insufficient for detecting early renal injury (Sanjeevani et al., 2014). Thus, novel biomarkers combining clinical sensitivity, specificity, and noninvasion are desired. circRNAs have been gradually employed in this field because of their universality and stability.

Inflammatory response including NF-κB pathways activation, interleukin 6 (IL-6) production are involved in the progression of AKI (Bonventre and Yang, 2011). ATF3 can block the activation of the NF-κB pathway and inhibit the release of IL-6 in AKI (Li et al., 2010). The expression of circ-0114427 is upregulated and can resist the early inflammatory state, by binding to miR-494 as an miRNA sponge to decrease ATF3 expression and further increase IL-6 expression (Cao et al., 2020). Similar, circVMA21 alleviated sepsis-associated AKI via regulating miR-9-3p/SMG1/inflammation and oxidative stress (Shi et al., 2020).

I/R induces the pathological damage and death of renal cells (Wang et al., 2017). Several circRNAs have been shown to be altered in IR–AKI model (Zhou et al., 2017). The increased circ-AKT3 promotes I/R injury progression via sponging to miR-144-5p to activate Wnt/β-catenin signal and regulating oxidative stress (Xu et al., 2020). While circYAP1 activates PI3K/AKT/mTOR pathway and secures HK-2 cells from I/R injury via sponging miR-21-5p (Huang et al., 2020). AKI also occurs in transplanted kidneys during or after the transplantation procedure itself (Munshi et al., 2011; Lameire et al., 2013). hsa_circ_0001334 is upregulated in patients who have acute kidney rejection, but it can return to normal levels when patients are successfully treated with an anti-rejection therapy (Kölling et al., 2019). A high ciRs-126 expression is connected with poor prognosis and an independent predictor of the 28 days survival of patients with AKI (Kölling et al., 2018).

Nephrotoxicity often occurs during disease diagnosis and treatment, and it accounts for approximately 20% of AKI cases (Uchino et al., 2005). Contrast-induced (CI) AKI is an acute renal insufficiency during disease diagnosis (Kellum and Lameire, 2013; Luo et al., 2017). Naglu is used to predict the prerenal development of AKI, and Gpnmb plays a protective role against AKI. It is reported that circ_0004153-rnomiR-144-3p-Gpnmb or Naglu, are validated in a CI-AKI rat model, and they are likely related to oxidative stress, drug metabolism through GO and KEGG pathway analyses (Cheng et al., 2019). Cisplatin chemotherapy is also a frequent cause of nephrotoxicity leading to AKI during disease treatment. A total of 224 upregulated circRNAs and 144 downregulated circRNAs, which are predominantly implicated in nucleic acid binding transcription and metabolic pathways, have been detected in cisplatin-treated mice through RNA sequencing analysis and bioinformatic analysis (Li et al., 2019).

Benefit from their structural stability and tissue specificity, circRNAs have great potential in the diagnosis and treatment of AKI at an early stage by changing the contents of urine and blood. More studies are needed to identify circRNAs specifically expressed during AKI development, and in-depth basic studies are warranted to assess their functions and improve AKI diagnosis and treatments.

## Circular RNAs in Chronic Kidney Diseases

CKD is characterized by a reduced glomerular filtration rate and increased urinary albumin excretion; it is an important cause of low life quality and death. Hypertension and diabetes, glomerulonephritis and unknown causes are common causes of CKD (Jha et al., 2013).

In a mouse model of hypertension-related kidney disease, circNr1h4 is significantly downregulated in kidneys. circNr1h4 sponges with miR-155-5p to decreases the expression of fatty acid reductase 1 (Far1), increasing reactive oxygen species, thereby causing damage to renal epithelial cells (Buchert et al., 2014; Lu et al., 2020). These findings may help develop new therapeutic strategies of targeting circRNAs for hypertension-related kidney diseases.

Diabetic nephropathy (DN) is characterized by the proliferation of mesangial cells and the accumulation of the extracellular matrix (Wang et al., 2018; Lu et al., 2019), and there is also increasing evidence of the role of the inflammatory response in developing DN (Yaribeygi et al., 2019). circ-AKT3 inhibited the extracellular matrix accumulation through modulating miR-296-3p/E-cadherin signals in diabetic nephropathy mesangial cells (Tang et al., 2020). circRNA_15698 is upregulated in db/db mice, and acts as an miR-185 sponge to regulate TGF-β1 expression, which promoting extracellular matrix-related protein synthesis (Hu et al., 2019). Likewise, circRNA_010383 expression is markedly downregulated, it promotes proteinuria and the accumulation ECM proteins and down-regulate the expression of transient receptor potential cation channel, subfamily C, member (TRPC1) leading to the aggravation of renal fibrosis in DN by acting as a sponge for miRNA-135a (Peng et al., 2020). circ_WBSCR17 is highly expressed in DN mice, it triggers fibrosis and in ammation through increasing the expression of SOX6 by targeting miR-185-5p (Li et al., 2020). What’s more, it has been reported that silencing cANRIL (circular antisense noncoding RNA in the INK4 locus) alleviates inflammatory responses and blocks NF-κB and JNK/p38 pathways by positively regulating miR-9 in LPS-induced CKD modle (Deng et al., 2019). While The increased circ_DLGAP4 sponges with miR-143 and activates ERBB3/NF-κB/MMP-2 to promote fibrosis of mesangial cells (Bai et al., 2020). circLRP6 was found to be upregulated in high glucose (HG)-treated mesangial cells, regulated HG-induced cell injure via sponging miR-205, upregulating HMGB1 and activating TLR4/NF-κB pathway (Chen et al., 2019). The regulatory effect of circRNAs in DN should be verified in human tissues further.

Idiopathic membranous nephropathy (IMN) and lupus nephritis (LN) belong to autoimmune diseases that have a long disease course and an impaired kidney function (Almaani et al., 2017; Cattran and Brenchley, 2017). circRNAs serve as a potential biomarker for the diagnosis of IMN and LN. Some intron-derived circRNAs are reduced in serum and urine exosomes of patients with IMN and may be involved in IMN pathogenesis (Ma et al., 2019). Plasma circRNA_002453 is considered to be a potential biomarker to assess the severity of renal involvement in patients with LN (Ouyang et al., 2018). Bioinformatic analysis has predicted that several circRNAs, which are significantly upregulated in LN, participate in regulating dendritic cell differentiation and MHC protein complex. circHLA-C is found to be significantly increased, and may serve as a sponge of miR-150, which promotes renal fibrosis by regulating fibrosis-associated genes (Zhou et al., 2013; Luan et al., 2018). These data suggest the possible roles of circRNAs in immune-related CKD development. However, large cohorts and *in vitro* and *in vivo* experiments are needed to clarify the detailed mechanism.

Kidney stone often manifests as urinary tract infection and pain, and is also a cause of CKD (Zeng et al., 2017). 58 upregulated and 87 downregulated circRNAs have been identified to reveal the significant differential expression in the pathogenesis of kidney stones (Cao et al., 2018). Further studies are also needed to determine the detailed mechanism on how circRNAs work in kidney stone growth and CKD development.

With multiple pathogenic factors, CKD is still a global public health issue. Even though circRNAs have attracted much attention in the development of CKDs, the specific mechanism and function of circRNAs are still needed to provide a precise and new perspective for diagnosing and treating CKD.

## Conclusion and Perspectives

In this review, multiple potential roles of circRNAs in RCC, AKI and CKD are summarized (Figure 2; Table 3), which enriching our understanding of the abundant circRNAs.

However, studies on the role of circRNAs in kidney-related diseases are still in the fledging period, most studies have elucidated biological phenomena mainly dependent on bioinformatic analyses but have not further systematically and experimentally explored the mechanisms of how circRNAs work in kidney-related diseases. There are some questions need to be discussed.

First, circRNAs are more stable, and they exist in specific tissues, giving them the potential of being ideal biomarkers for disease diagnosis. Current studies on circRNAs in RCC mainly focus on their effects on the biological function of RCC cells, and the expression of circRNAs was mainly detected in tumor tissues. But in AKI and CKD, circRNAs have been gradually identified as biomarkers combining clinical sensitivity, specificity and non-invasion to assess kidney function. The expression of circRNAs in blood or urine of patients with RCC may be a potential research topic to be used as a diagnostic basis for evaluating therapeutic efficacy. Even though most circRNAs are reported as biomarker, the correlation between the circRNAs levels and the degree of disease severity is not well analyzed. It needs more studies to confirm that whether circRNAs can return to normal level when patients with kidney-related diseases receive treatments.

Second, circRNAs can act as biomarkers in blood or urine, they settle in the cytoplasm once they are generated, how they are exported from the nucleus to the cytoplasm remains unclear. DExH/D-box helicase Hel25E participates in the extranuclear transport of circRNAs (>800 nt). The two human homologs of Hel25E are reported to participate the localization of circular RNA: UAP56 (DDX39B) contributes to the exportation of long (>1300 nt) circular RNAs, whereas URH49 (DDX39A) is necessary for the export of short (<400 nt) circular RNAs (Huang et al., 2018). Yet, how circRNAs between 400 and 800 nt are exported in humans is unknown.

Third, when circRNAs are exported, how they are degraded after they perform their functions is still ambiguous. Some circRNAs can be cleared by packaging into extracellular vesicles, such as exosomes (Lasda and Parker, 2016), which still act as biomarkers. CDR1as/ciRS-7 can be cleaved by Argonaute-2 (Ago2) to trigger transcript degradation (Hansen et al., 2013). circRNAs can be enriched in synapses and implicated in the development of neuronal differentiations (Rybak-Wolf et al., 2015; You et al., 2015), indicating the possibility of directional transmissions, some RNA endonucleases specifically exist in tissues to degrade certain circRNAs and cause their tissue specificity.

Furthermore, kidney-related diseases can interevolve to each other. Nephrectomy due to RCC may impair kidney function, AKI likely contributes to the development and progression of CKD, AKI is also one of the major complications in CKD (Uchino et al., 2005; He et al., 2017; Hassan et al., 2018). And cir-ATK3 takes part in RCC and AKI, whether exists a particular circRNA that regulates the progression of multiple kidney-related diseases simultaneously need to be confirmed.

Through continuous biological technology development and further circRNA exploration, circRNAs will eventually provide a new theoretical basis for conducting disease diagnosis, treatment in the coming years.

## Data Availability

The original contributions presented in the study are included in the article, further inquiries can be directed to the corresponding authors.

## Glossary

AKI: acute kidney injury;
AR: Androgen receptor;
CCRCC: clear cell renal cell carcinoma;
CDH1: cadherin 1, E-cadherin;
CDH2: cadherin 2, N-cadherin;
CI: Contrast-induced;
circRNAs: Circular RNAs;
ciRNAs: circular intron circRNAs;
CKD: chronic kidney disease;
DN: Diabetic nephropathy;
ecircRNAs: exonic circRNAs;
ECM: extracellular matrix;
EIciRNA: exon- and intron-derived or retained intron circRNAs;
EMT: Epithelial-to-mesenchymal transition;
ERBB3: Erb-b2 receptor tyrosine kinase 3;
ERβ: estrogen receptor beta;
Far1: fatty acid reductase 1;
FN1: Fibronectin 1;
FOXP4: forkhead-box P4;
HHLA2: Human endogenous retrovirus-H long terminal repeat-associating protein 2;
HIFs: hypoxia-induced factors;
HREs: hypoxia-responsive elements;
I/R: Ischemia/reperfusion;
IMN: Idiopathic membranous nephropathy;
IRF7: interferon regulatory factor 7;
LN: Lupus nephritis;
lncRNAs: long ncRNAs;
METTL14: methyltransferase-like 14;
miRNAs: MicroRNAs;
ncRNAs: noncoding RNAs;
RCC: renal cell carcinoma;
SMG1: serine/threonine-protein kinase 1;
SOX12: sex-determining region Y (SRY)-box 12;
TGF-β: Transforming growth factor β;
Tregs: regulatory T cells;
TRPC1: transient receptor potential cation channel 1;
ZEB2: Zinc finger E-box-binding homeobox 2.
